# Evaluation of Catfish Skin Gelatin-Based Edible Antimicrobial Coating with Lactic Acid and Potassium Sorbate on the Shelf Life and Quality of Fresh Catfish Fillets

**DOI:** 10.3390/gels12070584

**Published:** 2026-07-02

**Authors:** Katheryn Parraga, Hunter Songy, Evelyn Watts

**Affiliations:** 1 Department of Food Science, Purdue University, West Lafayette, IN 47907, USA; kparraga@purdue.edu; 2 School of Nutrition and Food Sciences, Louisiana State University Agricultural Center, Baton Rouge, LA 70803, USA

**Keywords:** catfish skin gelatin, fish fillets, shelf life, quality, gelatin characterization, organic acids

## Abstract

Edible coatings derived from fish by-products offer promising, sustainable approaches for seafood preservation. This study evaluated catfish skin gelatin as a carrier for antimicrobial agents to enhance microbiological stability, oxidative stability, and shelf life of fresh catfish fillets during refrigerated storage. Shelf life was defined as the storage period required for microbial and quality parameters to reach spoilage-associated limits. Treatments included control (C), lactic acid (LA), potassium sorbate (PS), gelatin (G), gelatin + LA (G+LA), and gelatin + PS (G+PS). Microbial quality (Aerobic Plate Count, psychrophiles, *Pseudomonas* spp., yeasts), physicochemical properties (pH, moisture, color), and lipid oxidation (TBARS) were assessed. Based on APC and psychrophiles, gelatin combined with antimicrobials was associated with extended shelf life relative to the control, with G+PS (15 days) and G+LA (12 days) compared to 9 days for untreated fillets, while LA and PS alone showed limited effects. All treatments maintained pH < 7.0, and color changes were minimal except for increased b* in LA treatments. TBARS increased during storage, particularly with LA, but remained below 5 mg MDA/kg. Overall, gelatin-based systems, particularly with PS or LA, offer a cost-effective, sustainable approach to potentially improve refrigerated fish quality while valorizing fish by-products. Further work is needed to optimize formulations and validate results under commercial conditions.

## 1. Introduction

Seafood represents a major economic sector in the United States, with the domestic seafood market valued at more than USD 25,000,000,000 annually, making it one of the largest seafood markets globally [1,2,3,4,5]. Despite this, nearly 47% of edible USA seafood is wasted, primarily at the consumer level, due to its short shelf life [6]. The high perishability of seafood is attributed to its rich nutrient profile, neutral pH, high moisture, and polyunsaturated fatty acids [7], which make it highly susceptible to microbial spoilage and oxidative changes [8,9]. The chemical composition of catfish flesh is characterized by high moisture and protein content, with moderate lipid levels that typically range from approximately 2–8% depending on production conditions. Although relatively lean, catfish lipids contain a substantial proportion of unsaturated fatty acids, including monounsaturated and polyunsaturated fatty acids, which are highly susceptible to oxidative degradation. These unsaturated lipids provide reactive sites for oxygen attack, leading to the formation of secondary oxidation products associated with rancid flavors and off-odors. Therefore, even moderate lipid levels can significantly influence quality deterioration, underscoring the importance of evaluating lipid oxidation in catfish during refrigerated storage [10,11,12,13]. Catfish fillets typically last 5–8 days under refrigeration [14,15], with microbial growth and lipid oxidation being the primary quality-degrading factors [14,16]. These factors lead to undesirable changes in color, odor, and texture, limiting fresh product availability and contributing to food waste.

Beyond consumer waste, fish processing generates substantial byproducts, 30–55% of catfish dressing weight, including frames, heads, viscera, and skin [17,18,19]. Valorization of these byproducts into functional ingredients offers sustainable solutions to reduce waste and improve profitability [20,21]. Among these, gelatin has gained attention as a versatile biopolymer for food applications, particularly in edible coatings and films that enhance product quality and reduce reliance on synthetic packaging [22,23]. Compared to bovine and porcine gelatin, fish gelatin typically exhibits lower gel strength and melting temperatures due to its reduced amino acid content, which can limit thermal stability at higher temperatures [24,25]. However, these properties are advantageous in refrigerated seafood systems, where increased flexibility and lower gelling temperatures facilitate coating formation without compromising texture [25]. Additionally, fish gelatin demonstrates comparable film-forming functionality while supporting the valorization of seafood by-products into value-added applications [25,26]. Fish-derived gelatin is increasingly favored over mammalian sources due to cultural and dietary restrictions [27,28]. Catfish represents the leading aquaculture species in the USA, both in terms of production volume and economic significance [29]. Additionally, catfish skin has been consistently demonstrated to be an effective raw material for gelatin extraction, yielding gelatins with functional and thermal properties comparable to commercial mammalian gelatin [19,27,30].

Edible coatings are thin, consumable layers applied to food surfaces to improve quality and extend shelf life by acting as barriers to mass transfer [31]. Gelatin-based coatings provide a physical barrier to moisture and oxygen while serving as carriers for antimicrobial agents, creating a synergistic effect that inhibits spoilage microorganisms and extends shelf life [32,33]. This functionality is partly attributed to antimicrobial peptides present in gelatin, which exhibit amphipathic properties enabling interaction with bacterial membranes, leading to pore formation and cell damage [34,35,36]. Recent studies have demonstrated the effectiveness of gelatin coatings combined with organic acids, essential oils, and preservatives in seafood preservation [8,37,38]. For example, Wei et al. [33] reported that gelatin coatings combined with vacuum packaging maintained golden pompano fillet quality for up to 14 days.

Organic acids, such as lactic acid, are widely used as antimicrobials in food systems. Their mechanism involves penetration of the bacterial cell membrane in the undissociated form, followed by intracellular dissociation, which disrupts pH homeostasis and depletes cellular energy [39]. Lactic acid has shown efficacy against Gram-negative bacteria, including *Enterobacteriaceae* and psychrotrophic species, though its effect on yeasts is limited [14]. More recently, lactic acid treatment of catfish fillets has been shown to significantly suppress microbial growth during refrigerated storage, maintaining bacterial counts near 4–5 Log CFU/g compared with 6 Log CFU/g in untreated controls over similar storage periods [40]. Similarly, potassium sorbate, a widely used preservative, inhibits fungal and bacterial growth by interfering with microbial enzyme systems [41]. Gandotra et al. [42] demonstrated that dipping silver carp fillets in potassium sorbate extended shelf life significantly, with treated samples showing only 1.27 Log CFU/g compared to 6 Log CFU/g in controls after 20 days. Lactic acid and potassium sorbate are approved food ingredients permitted for use in fish and fishery products under current good manufacturing practice (GMP), without specified maximum use levels (21 CFR § 184.1061; 21 CFR § 182.3640).

Recent advances in edible coatings emphasize active packaging strategies incorporating natural antimicrobials and controlled-release systems to improve food safety and sustainability [22,43]. Gelatin-based coatings enriched with lactic acid and potassium sorbate represent a promising approach for seafood preservation, combining physical and chemical hurdles to inhibit microbial growth while maintaining desirable physicochemical properties. However, despite the demonstrated effectiveness of gelatin coatings and the individual antimicrobial properties of lactic acid and potassium sorbate, limited information exists on their combined use within fish-derived gelatin systems, particularly for freshwater species such as catfish. In addition, the potential interactions between these treatments and their simultaneous effects on microbial inhibition and lipid oxidation have not been comprehensively evaluated. While these combinations have been studied in a number of food products, their application to catfish fillets remains unexplored.

Therefore, the objective of this study was to evaluate the effectiveness of catfish skin gelatin coatings combined with lactic acid and potassium sorbate in enhancing the microbiological stability, oxidative stability, and shelf life of fresh catfish fillets during refrigerated storage. Specifically, the study aimed to assess the impact of these treatments on microbial growth dynamics, physicochemical properties, and lipid oxidation to determine their potential as a cost-effective strategy for improving the quality and safety of catfish fillets.

## 2. Results and Discussion

### 2.1. Catfish Skin Gelatin Characterization

The physical and functional properties of catfish skin gelatin were evaluated, including amino acid composition, gel strength, melting temperature, setting temperature and setting time.

Amino acid analysis revealed glycine as the most abundant residue (392 residues per 1000), followed by alanine (129) and proline (113). Glycine content was notably higher than African Catfish (250), Tilapia (347), Megrim (350), and Cod (344), while proline remained similar across species (113 vs. 106–119) [24,44,45] (Table 1).

The gel strength of the extracted catfish skin gelatin was 376.20 ± 12.17 g, which is higher than previously reported for African Catfish (286.71 g) [44] and other freshwater species such as snakehead (*Channa striatus*; 311 g), pangasius (*Pangasius sutchi*; 324 g), and catfish (*Clarias batrachus*; 278 g) [46]. Tilapia exhibited the highest gel strength among freshwater species at 487 g [46]. The observed value also exceeds the typical range for commercial mammalian gelatin (200–300 g) [47,48], indicating strong gel-forming capability. The superior gel strength observed can be attributed primarily to amino acid composition. Glycine and proline are essential for stabilizing the collagen triple helix and forming strong gel networks [49,50]. Glycine, the most abundant amino acid in gelatin, promotes tight molecular packing and hydrogen bonding, while proline contributes to rigidity and thermal stability [51]. The notably higher glycine content observed in Channel Catfish gelatin (392 residues/1000) compared to other species likely promotes a more compact triple-helix structure, enhancing intermolecular interactions and contributing to the high gel strength observed. Although proline levels were similar across species, the elevated alanine content in catfish may further support gel network stabilization through hydrophobic interactions, suggesting that variations in multiple amino acids influence functional properties beyond amino acid content alone [45,48,52]. Higher gel strength is directly associated with improved mechanical stability of gelatin matrices, resulting in more cohesive and elastic networks that resist deformation under stress. This characteristic is particularly important for edible coating applications, as stronger gels enhance film integrity and reduce structural breakdown during handling and storage. Additionally, increased gel strength contributes to improved barrier properties by forming denser polymer networks that limit oxygen and moisture transfer, thereby enhancing protection against oxidative reactions and microbial proliferation in coated food systems [45,48,52].

The melting temperature of catfish skin gelatin was determined to be 31.46 ± 0.35 °C, which exceeds the conventional range for warm-water fish gelatin (20–29 °C) [45] and African catfish (25.7 °C) [53]. This value is comparable to commercial gelatin standards [47,48], reflecting enhanced thermal stability. A melting point above 30 °C ensures that the gelatin remains solid under typical ambient conditions, thereby minimizing risks associated with premature melting during handling, storage, and transportation. Such thermal stability is essential for diverse applications including edible coatings, encapsulation, and confectionery, where maintaining structural integrity at room temperature is vital [47]. Additionally, elevated melting points are associated with higher proline content, which contributes to both gel rigidity and heat resistance [54]. The observed increase in gel strength and melting point in this study is likely influenced by the combined presence of high glycine content together with sufficient proline, which collectively enhance molecular packing and thermal stability of the gelatin network [45].

Setting temperature and time also provide insight into gelation behavior. The gelatin setting temperature (17.63 ± 0.60 °C) falls within the typical range for warm-water fish species (15–22 °C; [45]) and is comparable to common carp (17.9 °C) and African Catfish (19 °C) [47,55]. However, the gel setting time (285.6 ± 10.2 s) was significantly longer than previously reported for other species, such as common carp (103.19 s) and African Catfish (106.90 s). Longer setting times may be associated with higher proportions of β and γ polypeptide chains, which can influence gelation kinetics by reducing molecular mobility and potentially delaying network formation [47,48]. While extended setting times may pose challenges for certain industrial processes, they do not diminish the overall gel strength or thermal stability of the final product.

### 2.2. Evaluation of Potassium Sorbate, Lactic Acid, Gelatin, Gelatin with Potassium Sorbate, and Gelatin with Lactic Acid

Shelf life was defined as the storage period during which fillets remained within acceptable microbiological and quality limits, with termination occurring when any individual criterion was met, including microbial counts reaching spoilage levels or quality parameters (TBARS, pH, or color) exceeding acceptable thresholds, consistent with ICMSF recommendations for combining microbiological and quality criteria [56].

#### 2.2.1. Microbial Analyses

**Figure 1:**
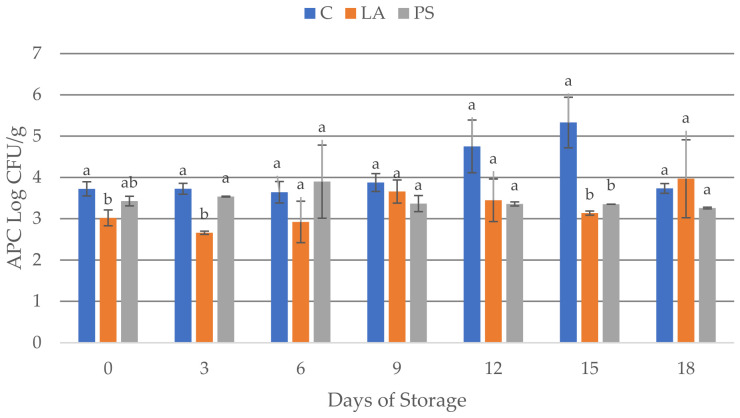
Aerobic Plate Counts (APC) during 18-day shelf life study of catfish fillets comparing treatments: untreated (C), Lactic acid (LA), and Potassium sorbate (PS). Log CFU/g: Logarithmic Colony-Forming Units per gram of sample. Mean values labeled with different letters on the same day are statistically significant (*p* < 0.05).

**Figure 2:**
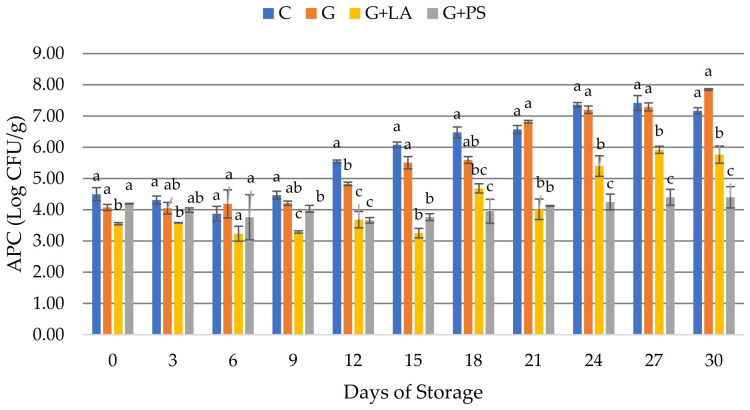
Aerobic Plate Counts (APC) during 30-day shelf life study of catfish fillets comparing treatments: untreated (C), Gelatin (G), Gelatin + Lactic acid (G+LA), and Gelatin + Potassium sorbate (G+PS). Log CFU/g: Logarithmic Colony-Forming Units per gram of sample. Mean values labeled with different letters on the same day are statistically significant (*p* < 0.05).

Aerobic plate count (APC) is a widely used indicator of overall microbial quality and shelf life in fresh fish products, as it reflects the presence of spoilage organisms and potential safety concerns. A threshold of 6 Log CFU/g is generally considered the upper limit for acceptable microbial quality in fresh fish [57]. In the first experiment, APC results indicated no significant reduction in the initial microbial load of catfish fillets treated with lactic acid (LA) or potassium sorbate (PS) compared to the untreated control (C), with counts of 3.02 ± 0.19, 3.43 ± 0.12, and 3.72 ± 0.17 Log CFU/g, respectively. However, by day 3, LA-treated fillets exhibited significantly lower APC compared to C and PS. Throughout storage, APC remained stable for LA and PS, whereas C showed a significant increase by day 15 (*p* < 0.05) (Figure 1). In the second experiment, initial APC values for C, gelatin (G), gelatin plus potassium sorbate (G+PS), and gelatin plus lactic acid (G+LA) were 4.50 ± 0.21, 4.08 ± 0.10, 4.20 ± 0.01, and 3.55 ± 0.03 Log CFU/g, respectively, with G+LA significantly lower than the other treatments. Over time, the 6 Log CFU/g threshold was reached first by C (6.08 ± 0.09 Log CFU/g) on day 15, followed by G (6.82 ± 0.04 Log CFU/g) after day 18, and G+LA (5.92 ± 0.11 Log CFU/g) on day 27, while G+PS maintained stable counts throughout storage (Figure 2).

**Figure 3:**
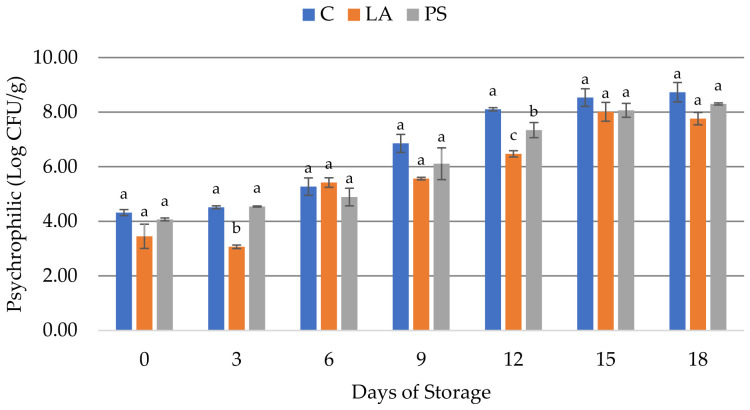
Psychrophilic counts during 18-day shelf life study of catfish fillets comparing treatments: untreated (C), Lactic acid (LA), and Potassium sorbate (PS). Log CFU/g: Logarithmic Colony Forming Units per gram of sample. Mean values labeled with different letters on the same day are statistically significant (*p* < 0.05).

**Figure 4:**
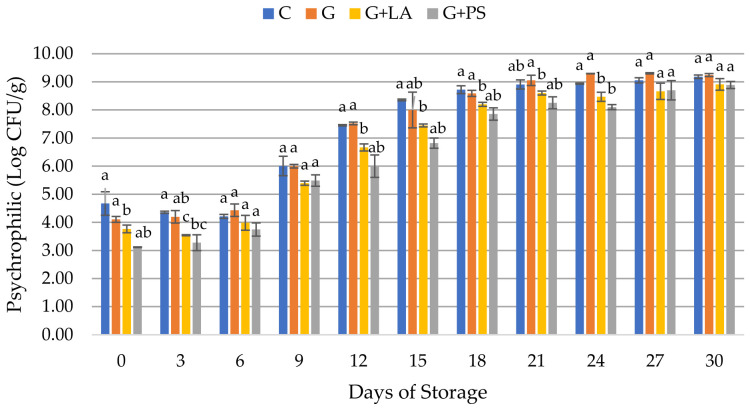
Psychrophilic counts during 30-day shelf life study of catfish fillets comparing treatments: untreated (C), Gelatin (G), Gelatin + Lactic acid (G+LA), and Gelatin + Potassium sorbate (G+PS). Log CFU/g: Logarithmic Colony-Forming Units per gram of sample. Mean values labeled with different letters on the same day are statistically significant (*p* < 0.05).

Psychrophilic bacteria, key indicators of spoilage in fresh fish due to their ability to grow at refrigeration temperatures and dominate the microbiota under chilled storage conditions, thereby driving quality deterioration, were monitored using a 7 Log CFU/g limit recommended by the International Commission on Microbiological Specifications for Foods [58]. In experiment one, initial counts for C, LA, and PS were similar to APC (3.72 ± 0.17, 3.02 ± 0.19, and 3.43 ± 0.12 Log CFU/g). All groups showed an increasing trend, with C and PS exceeding the 7 Log CFU/g threshold by day 12 (8.11 ± 0.06 and 7.34 ± 0.28 Log CFU/g, respectively), followed by LA on day 15 (8.01 ± 0.34 Log CFU/g). The LA-treated group exhibited significantly lower counts than C and PS on days 3 and 12 (Figure 3). In experiment two, G+LA showed an initial significant reduction (3.77 ± 0.14 Log CFU/g) compared to C, G, and G+PS (4.67 ± 0.42, 4.11 ± 0.10, and 3.12 ± 0.01 Log CFU/g). All treatments increased over time, with C and G reaching 7 Log CFU/g on day 12 (7.46 ± 0.02 and 7.52 ± 0.05), G+LA on day 15 (7.45 ± 0.05), and G+PS on day 18 (7.86 ± 0.22) (Figure 4). G+LA consistently showed lower counts than C and G on days 0, 3, 12, 18, and 24, while G+PS was significantly lower only on days 0 and 24. Psychrophilic bacteria reached the spoilage threshold earlier than APC.

*Pseudomonas* spp., another major spoilage indicator in fresh fish and a Gram-negative psychrotrophic bacterium that grows under refrigeration [59], was evaluated using the same 7 Log CFU/g threshold [58]. In experiment one, initial counts did not differ significantly among C, LA, and PS (3.26 ± 0.17, 3.15 ± 0.05, and 2.84 ± 0.16 Log CFU/g). All groups increased over time, with C and PS surpassing the threshold on day 12 (7.30 ± 0.03 and 7.05 ± 0.11), followed by LA on day 15 (7.30 ± 0.20). The LA-treated group showed significantly lower counts on days 3, 6, and 12. In experiment two, initial counts were similar across treatments (C 2.42 ± 0.06, G 2.81 ± 0.05, G+LA 2.65 ± 0.18, and G+PS 2.20 ± 0.08 Log CFU/g). All groups increased during storage, with C and G exceeding the threshold on day 12 (7.16 ± 0.13 and 7.01 ± 0.11), G+PS on day 15 (7.16 ± 0.06), and G+LA on day 18 (7.26 ± 0.16).

Yeasts, which can accelerate deterioration and cause sensory rejection at levels of 7–8 Log CFU/g [60], were also monitored. In experiment one, no significant differences were observed among treatments at any storage day. Initial counts were 2.94 ± 0.11, 2.73 ± 0.55, and 2.63 ± 0.29 Log CFU/g for C, LA, and PS, respectively, increasing to 5.63 ± 0.06, 5.46 ± 0.52, and 5.84 ± 0.17 by day 18. Similarly, in experiment two, initial counts did not differ among treatments (C 2.72 ± 0.14, G 2.10 ± 0.25, G+LA 2.28 ± 0.07, and G+PS 2.45 ± 0.01), and all groups showed an increasing trend over 30 days, reaching 5.98 ± 0.09, 5.63 ± 0.31, 6.60 ± 0.08, and 6.07 ± 0.09 Log CFU/g for C, G, G+LA, and G+PS, respectively.

It should be noted that the two experiments were conducted as independent trials using different raw material batches, which likely contributed to variation in initial microbial loads and spoilage kinetics. Such variability is typical in fresh seafood systems due to differences in intrinsic microbiota, environmental exposure, and handling conditions, all of which influence microbial composition and subsequent spoilage behavior [61,62]. However, consistent treatment effects observed across both trials suggest that the gelatin-based treatments maintained similar trends in delaying microbial growth under refrigerated storage. The initial reduction observed with LA treatment aligns with previous studies reporting its antimicrobial efficacy. Farid et al. [14] and Masniyom & Benjama [63] found significant reductions of approximately 1 Log CFU/g on the first day of treatment when LA was applied to seafood products, consistent with the early inhibitory effect observed in this study. The extended shelf life achieved with gelatin combined with LA surpasses the 6-day shelf life reported by Kazemi & Rezaei for rainbow trout slices treated with gelatin–alginate films, highlighting the potential of LA-gelatin systems for improved preservation [64]. Similarly, Jiang et al. [8] demonstrated that catfish gelatin combined with PS extended shrimp shelf life to 22 days, supporting our findings that PS, particularly when incorporated into gelatin coatings, can stabilize microbial growth and delay spoilage.

The antimicrobial effectiveness of LA and PS can be attributed to their distinct mechanisms of action. Lactic acid lowers the pH of the food surface and disrupts microbial cell membranes, creating an environment unfavorable for spoilage and pathogenic bacteria [65,66]. It also interferes with enzymatic activity essential for microbial metabolism, which explains the initial reduction in microbial counts and delayed spoilage in LA-treated fillets. Potassium sorbate, the potassium salt of sorbic acid, inhibits microbial growth primarily through the undissociated acid, which penetrates cell membranes, disrupts intracellular pH homeostasis, and interferes with enzymes involved in energy metabolism, resulting in effective suppression of molds, yeasts, and selected bacteria [67,68]. Its antimicrobial efficacy is strongly pH-dependent, with greater activity under acidic conditions that favor membrane penetration and metabolic disruption, making potassium sorbate particularly effective for acidified seafood systems [69,70].

The incorporation of these antimicrobials into gelatin coatings further enhances their effectiveness. According to literature, gelatin forms a semi-permeable barrier that reduces oxygen transfer and moisture loss, slowing lipid oxidation and microbial proliferation [71,72]. Furthermore, when combined with LA or PS, gelatin may act as a carrier facilitating prolonged contact of the antimicrobial agents with the product surface and potentially enabling controlled release over time [73]. This synergistic effect explains the extended shelf life observed with G+LA and G+PS treatments compared to single-agent applications. Recent studies further support the effectiveness of gelatin-based coatings as active systems capable of incorporating antimicrobial and antioxidant agents, enhancing their functionality beyond passive barrier properties [71,74]. Similar findings have been reported for shrimp and other seafood products, where gelatin-based coatings infused with organic acids or sorbates significantly delayed psychrophilic and *Pseudomonas* growth [8,72].

These results also confirm that psychrophilic bacteria reached spoilage thresholds before APC, as previously reported for fresh shrimp [8,72], reinforcing their role as primary indicators of quality loss. The effectiveness of organic acids and preservatives such as PS in extending seafood shelf life has been widely documented [14,42,63]. However, consistent with Farid et al. [14], LA and PS were less effective against yeasts, which continued to increase throughout storage. This trend is consistent with recent literature identifying psychrotrophic bacteria such as *Pseudomonas* and *Shewanella*, along with yeasts, as dominant spoilage organisms in refrigerated fish products. These microorganisms are well adapted to low temperatures and high-moisture environments, making them key drivers of quality deterioration despite the presence of antimicrobial treatments [71,75,76,77]. Furthermore, Gençcelep et al. [78] observed a 2 Log CFU/g increase in yeasts and mold counts in pearl mullet fillets treated with potassium sorbate during a 12-day study. Similarly, Kazemi & Rezaei [64] reported rapid *Pseudomonas* spp. growth in gelatin-alginate films, reaching 8.71 Log CFU/g by day 15 [64], comparable to the increasing trend observed in our study.

The lack of significant differences among treatments suggests that LA and PS, either alone or incorporated into gelatin, may have had limited inhibitory effects on yeasts growth, likely due to the inherent tolerance of spoilage yeasts to weak acids and preservatives. Although yeasts count increased during storage, this trend is consistent with previous reports indicating that yeasts are commonly associated with refrigerated seafood and can persist under chilled conditions, and levels remained below those typically associated with sensory rejection [79,80].

While the microbiological parameters evaluated in this study (APC, psychrotrophic counts, *Pseudomonas* spp., and yeasts) are widely recognized indicators of spoilage in aerobically stored fresh fish, it is important to acknowledge that the microbiological panel was not exhaustive. Additional spoilage-associated groups, such as lactic acid bacteria, H_2_S-producing bacteria (e.g., *Shewanella* spp.), and Enterobacteriaceae, were not included. These microorganisms may contribute to spoilage dynamics under certain storage or treatment conditions. Therefore, the results should be interpreted as reflecting the behavior of the specific microbial groups monitored, rather than a comprehensive characterization of the entire spoilage microbiota.

#### 2.2.2. Physical and Chemical Analyses

Moisture content in catfish fillets remained generally stable during refrigerated storage, averaging 75–80%, consistent with previous findings for fresh catfish fillets [59]. In the first experiment, LA-treated fillets showed significantly lower initial moisture (63.6 ± 2.7%) compared to C (81.4 ± 0.7%) and PS (79.6 ± 2.0%) (*p* < 0.05). A similar trend persisted on day 6, but differences disappeared thereafter. LA exhibited an increasing trend from 63.6% on day 0 to 79.9% on day 15, while C and PS remained stable. In the second experiment, initial moisture did not differ significantly among treatments (C 80.9%, G 79.0%, G+LA 79.0%, G+PS 76.6%), and remained stable except for C, which decreased slightly on day 21.

The initial moisture reduction in LA-treated fillets likely resulted from the osmotic effect of lactic acid, which can alter ionic balance and protein structure, reducing water-holding capacity [14,63]. Over time, moisture recovery suggests redistribution within muscle tissue as proteins equilibrate under refrigeration, a phenomenon previously reported in fish treated with organic acids [42]. Gelatin-based coatings maintained moisture effectively, as mentioned in the literature, act as semi-permeable barriers that reduce water migration and evaporation while serving as carriers for antimicrobials [71,72].

Moisture is critical for microbial growth since fish muscle contains over 80% water, providing an ideal environment for spoilage organisms [59]. The initial reduction in LA-treated fillets may have contributed to lower microbial counts early in storage, as reduced water activity limits bacterial proliferation. However, as moisture increased, microbial growth resumed, underscoring the importance of combining LA with barrier technologies such as gelatin coatings to sustain antimicrobial efficacy and moisture control throughout storage.

The pH is an important indicator of spoilage in fresh fish; a pH of 6.8 to 7.0 post-mortem has been used as a limit of acceptability in fresh fish [59,81]. The pH values were monitored as an indicator of freshness during refrigerated storage. In the first experiment, PS initially exhibited a significantly lower pH (6.68 ± 0.14) than C (6.87 ± 0.10), while LA started at 6.85 ± 0.13. Over time, C and LA decreased to 6.61 ± 0.11 and 5.99 ± 0.20, respectively, whereas PS increased to 6.97 ± 0.20 by day 15. The LA-treated group maintained significantly lower pH than other treatments from day 9 onward (*p* < 0.05). In the second experiment, initial pH values were similar among treatments (6.69–6.98). All groups showed gradual decreases, with G+LA reaching 6.43 ± 0.18 and G+PS 6.64 ± 0.10 by day 30. Across both experiments, all treatments remained below the spoilage threshold of 7.0 [59,81]. The effects of pH reduction induced by LA and G+LA treatments are closely linked to other quality changes observed in this study. Lower pH can promote protein denaturation and structural disruption of muscle tissue, which in turn facilitates the release of bound water, pigments, and pro-oxidant components such as heme iron. These changes contribute to both microbial inhibition in early storage and the progression of physicochemical deterioration over time [79,82].

The marked pH reduction in LA-treated fillets reflects its antimicrobial mechanism: lactic acid lowers surface pH, creating unfavorable conditions for microbial growth and enhancing efficacy through increased undissociated acid molecules at lower pH [83,84,85]. This has likely contributed to reduced microbial counts early in storage. Potassium sorbate also exhibits pH-dependent activity, being most effective under acidic conditions where sorbic acid predominates [86]. Although PS-treated fillets showed slight pH increases, initial acidity likely supported its antifungal effect [87].

Combining LA or PS with gelatin coatings further improved preservation, which, according to the literature, add a physical barrier that limit oxygen transfer and moisture loss while ensuring controlled antimicrobial release [71,72]. Similar synergistic effects have been reported in seafood coatings incorporating organic acids or sorbates [33,88].

Color parameters (L*, a*, b*) were monitored as indicators of visual quality during refrigerated storage. In the first experiment, initial L* values showed no significant differences among treatments: C (65.7 ± 2.5), LA (62.5 ± 3.3), and PS (62.6 ± 3.2). Across storage, L* remained stable, though PS appeared darker than LA at later time points. The a* values showed no differences among treatments, remaining between −0.2 ± 1.8 and 2.4 ± 2.6. For b*, LA exhibited significantly lower initial values (3.6 ± 2.0) compared to C (1.9 ± 2.2) and PS (2.0 ± 1.6), and similar differences persisted throughout storage. LA-treated fillets reached 7.5 ± 3.5 by day 18, indicating increased yellowness. In the second experiment, initial L* values were similar among treatments: C (63.9 ± 1.1), G (64.6 ± 1.4), G+LA (64.4 ± 0.4), and G+PS (65.8 ± 1.3). On day 15, G+LA (63.7 ± 1.4) was significantly lower than C (67.3 ± 2.3). a* values remained stable (−1.13 ± 1.26 to 1.67 ± 1.51), while G+LA exhibited higher b* values on multiple days, suggesting amplified yellow tones. This increase in yellowness is mechanistically associated with acid-induced protein denaturation and enhanced lipid oxidation. As structural proteins are destabilized at lower pH, lipid–protein interactions are altered, exposing lipids to oxidation and leading to the formation of secondary oxidation products that contribute to yellow discoloration [89,90]. Additionally, LA accelerates lipid oxidation, generating secondary products such as aldehydes that impart yellow coloration [71]. Gelatin coatings combined with LA may prolong this effect by retaining surface moisture and facilitating controlled acid release.

Conversely, PS-treated fillets maintained relatively stable color because potassium sorbate acts primarily as an antifungal agent, without significantly altering muscle pH or protein structure [87]. Its neutral nature minimizes pigment disruption and oxidative reactions, explaining the absence of yellowing. Gelatin alone showed negligible impact, consistent with its role as a transparent barrier [33].

**Figure 5:**
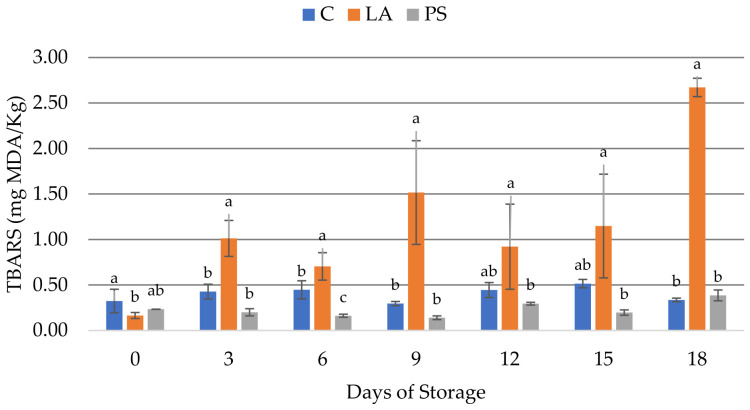
Thiobarbituric-acid-reactive-substances (TBARS, mg malonaldehyde (MDA) equivalent/Kg of tissue) values during an 18-day shelf life study of catfish fillets comparing treatments: untreated (C), Lactic acid (LA), and Potassium sorbate (PS). Mean values labeled with different letters on the same day are statistically significant (*p* < 0.05).

**Figure 6:**
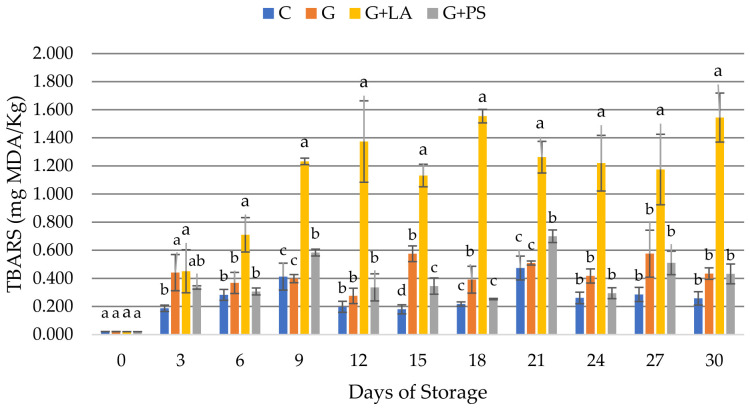
Thiobarbituric-acid-reactive-substances (TBARS, mg malonaldehyde (MDA) equivalent/Kg of tissue) values during a 30-day shelf life study of catfish fillets comparing treatments: untreated (C), Gelatin (G), Gelatin + lactic acid (G+LA), and Gelatin + Potassium sorbate (G+PS). Mean values labeled with different letters on the same day are statistically significant (*p* < 0.05).

Thiobarbituric acid reactive substances (TBARS), expressed as mg malondialdehyde (MDA)/kg, were used to assess lipid oxidation during storage. According to Bonilla et al. [91] an acceptable level of MDA equivalent/kg is 5 mg. In the first experiment, LA-treated fillets showed significantly lower initial TBARS (0.16 ± 0.03 mg MDA/kg) than C (0.32 ± 0.13 mg MDA/kg), but similar to PS (0.23 ± 0.00 mg MDA/kg). After day 3, LA exhibited the highest TBARS values, increasing steadily, while C remained stable and PS showed minimal change (Figure 5). In the second experiment, initial TBARS values were similar across treatments (≈0.022 mg MDA/kg). From day 6 onward, G+LA increased sharply, reaching 1.545 ± 0.174 mg MDA/kg by the end of storage, compared to C (0.258 ± 0.048 mg MDA/kg), G (0.434 ± 0.041 mg MDA/kg), and G+PS (0.432 ± 0.070 mg MDA/kg) (Figure 6). None exceeded the 5 mg MDA/kg threshold for quality loss [91].

Evaluation of TBARS is a widely accepted indicator of secondary lipid oxidation in fish, reflecting malondialdehyde formation from hydroperoxide breakdown [73,74]. The initial low TBARS values in LA treatments suggest short-term inhibition of antioxidation; however, subsequent increases indicate a pro-oxidative effect over storage. This is likely due to acid-induced protein denaturation and membrane disruption, which promotes the release of pro-oxidants such as heme iron and enhances oxygen diffusion into the muscle matrix. The resulting increase in free iron availability can accelerate lipid oxidation through catalytic reactions. The greater increase observed in G+LA treatments may be attributed to prolonged acid exposure from the gelatin matrix, intensifying tissue disruption and oxidative processes. Similar trends have been reported with organic acid treatments in seafood systems [22,75].

Conversely, PS and G+PS exhibited minimal TBARS increases, consistent with sorbate’s antioxidant properties [71,87]. Gelatin alone provided moderate protection by limiting oxygen exposure. These findings highlight the need to combine LA with antioxidants or packaging strategies to balance microbial control and oxidative stability. Overall, these findings align with current advances in active edible coatings and seafood preservation strategies, reinforcing the role of biopolymer-based systems such as gelatin in improving shelf life through combined barriers and antimicrobial effects under refrigerated storage conditions [22,92]. The stability of gelatin-based coatings during storage may be influenced by proteolytic activity from psychrotrophic spoilage microorganisms, such as Pseudomonas and Shewanella, which are known to produce extracellular proteases capable of degrading muscle and protein matrices. This enzymatic activity could affect the structural integrity of the gelatin coating, potentially altering its barrier properties and the release behavior of incorporated antimicrobial compounds over time. Such interactions between spoilage microbiota and protein-based coatings are consistent with previously reported observations in seafood systems, where microbial proteolysis plays a key role in quality deterioration and matrix modification [62,93].

## 3. Conclusions

Catfish skin gelatin demonstrated promising functional properties, including high gel strength, elevated melting point, and suitable setting behavior, which supports its potential as an alternative to conventional mammalian gelatin in food applications. These characteristics, combined with the opportunity to valorize seafood byproducts, highlight its relevance as a sustainable ingredient for food systems [20,21].

This study evaluated the effectiveness of catfish skin gelatin combined with lactic acid (LA) or potassium sorbate (PS) in improving microbiological stability, oxidative stability, and shelf life of refrigerated catfish fillets. Based on APC and psychrophiles, the results suggest that gelatin-based treatments, particularly when combined with PS or LA, may contribute to improved microbial control and extended shelf life relative to the untreated control. Among the treatments, gelatin combined with PS showed the greatest delay in microbial growth, followed by gelatin with LA, while gelatin alone provided limited improvement. These effects are likely associated with the combined barrier properties of gelatin and the antimicrobial activity of LA and PS; however, the extent of synergistic interactions may depend on formulation and storage conditions.

The integrated evaluation of microbial (APC, psychrotrophic bacteria, *Pseudomonas* spp., and yeasts), physicochemical, and oxidative parameters indicates that these treatments may help maintain product quality during refrigerated storage. However, the observed increase in lipid oxidation in LA-treated samples highlights the need to balance antimicrobial efficacy with oxidative stability and suggests that incorporation of antioxidants may be necessary for optimized formulations.

This study was conducted under controlled laboratory conditions using iced storage, which is a common practice in the United States seafood industry. However, storage conditions, handling practices, and temperature control can vary across global supply chains, which may influence microbial dynamics and treatment effectiveness. Therefore, caution should be exercised when extrapolating these findings to other production and distribution systems.

Additional limitations include the absence of sensory and texture evaluations, which are important determinants of consumer acceptance. Future research should focus on optimizing antimicrobial concentrations, incorporating natural antioxidants, and validating these findings under commercial conditions, including different storage systems. Further investigation using multivariate approaches may also help clarify the relationships among microbial, physicochemical, and oxidative changes.

Overall, catfish skin gelatin, particularly when combined with LA or PS, shows potential as a value-added strategy for enhancing the quality and shelf life of refrigerated catfish fillets. However, these findings should be considered preliminary, and further work is needed to confirm their applicability under diverse processing and storage conditions.

## 4. Materials and Methods

### 4.1. Catfish Skin Collection and Gelatin Preparation

Fresh farm-raised Channel Catfish (*Ictalurus punctatus*) skins were obtained from a local processor (Breaux Bridge, LA, USA). Skins were transported in ice to the LSU Baton Rouge Campus to the Seafood Quality Laboratory. The skins were vacuum-packed and frozen until extraction. Skins were stored at −20 °C for no longer than two months before extraction of the gelatin. The gelatin was extracted following the method described by Yang et al. [94] with slight modifications. Before the extraction process, the skins were thawed at 4 °C for at least 20 h. Then, the skins were cut into 2 cm strips followed by a washing step using tap water at 4 °C for 10 min (1:6 *w*/*v* ratio). This step was repeated three times. The cleaned skin strips were drained using a plastic colander strainer (Good Grip 3 QT 13.7 × 27.9 cm., OXO, New York, NY, USA) for five min and then pressed against a flat surface to remove most of the water. The fish skins were placed in a 3.5 gallon bucket and treated with a solution of 0.20 M NaOH for 84 min (VWR International, LLC, Radnor, PA, USA) (1:6 *w*/*v* ratio). Skin strips were then drained using the colander strainer, pressed against a flat surface, and rinsed with tap water (1:6 *w*/*v*). The NaOH treatment and the rinse step were repeated two times. Then, a solution of 0.115 M acetic acid (VWR International, LLC) (1:6 *w*/*v*) was used to treat the samples for 60 min, followed by draining using the colander strainer, pressed against a flat surface, and a rinse step with tap water (1:6 *w*/*v*) three times. All the reagents used were of analytical grade, and all the solutions were kept at 4 °C. After these steps, deionized water (1:4 *w*/*v*) was added to the skins, and the containers were covered with aluminum foil and plastic wrap. Containers were placed in a water bath at 55 °C for 180 min (the water bath conditions were created using an ice chest, 110 qt. (Igloo, Katy, TX, USA) with an immersion circulator (Anova Precision^®^ Cooker, San Francisco, CA, USA). To obtain the final gelatin solution, a filtration step was performed using four layers of cheesecloth (S. Ceng, grade 90, 36 Sq. feet, unbleached cotton fabric, ultrafine cheese cloth for cooking, Ces Centre, Singapore), followed by a lyophilization step (Genesis Pilot Lyophilizer, VirTis™ SP SCIENTIFIC, SP Industries, Warminster, PA, USA). Once the gelatin liquid was filtered, it was measured into 1.5 L aliquots and placed in freeze-dry trays. The trays were placed into a −80 °C freezer and allowed to solidify for 24 h. Once frozen, trays were placed in a VirTis™ SP Scientific Genesis Pilot Lyophilizer (Virtis, an SP Industries Company, Gardiner, NY, USA) and allowed to thoroughly dry for a period of 5 days. Dried gelatin was scraped out of the trays, placed in vacuum bags (3 mm, 55.3 cc/m^2^/day at 24 °C Oxygen transmission rate, Acadia Scales & Equipment, Opelousas, LA, USA), sealed, and placed in the −20 °C freezer (Environmental Growth Chambers, Chagrin Falls, OH, USA) until it was needed for characterization and shelf-life studies.

### 4.2. Catfish Skin Gelatin Characterization

Various analyses were undertaken to characterize the gelatin extracted from catfish skin. These analyses include amino acid analysis, gel strength, melting point, setting temperature, and setting time. Each of these parameters provides valuable insight into the functional properties and potential applications of gelatin. All analyses were run in six replicates.

Amino acid analysis was performed using a protocol adapted from the Pico•Tag Amino Acid Analysis Column Care and Use Manual (Waters Corporation, Milford, MA, USA). Approximately 1 g of gel was weighed into a hydrolysis tube and combined with 7 mL of 6 N HCl containing 0.25% phenol (VWR International, LLC). The tube was frozen, evacuated under vacuum for 2 min, and sealed; this freeze–vacuum cycle was repeated three times. Samples were hydrolyzed at 110 °C for 24 h. The hydrolysate was filtered, transferred to a 50 mL volumetric flask, and brought to volume. A 10 µL aliquot was mixed with 20 µL of norleucine (2.5 µmol/mL) and dried. The residue was derivatized with 100 µL of PITC solution (EtOH:water:PITC:triethylamine = 7:1:1:1) for 30 min and freeze-dried. The derivatized sample was dissolved in 1 mL of buffer (140 mM sodium acetate, 0.05% triethylamine, pH 6.40 adjusted with glacial acetic acid, plus 60 mL/L acetonitrile) and filtered through a 0.2 µm membrane for injection.

Pierce Amino Acid Standard H (ThermoFisher Scientific, Waltham, MA, USA) was used for calibration. The standard comprised 2.5 µmol/mL each of alanine, arginine, aspartic acid, glutamic acid, glycine, histidine, isoleucine, leucine, lysine, methionine, phenylalanine, proline, serine, threonine, tyrosine, and valine, along with 1.25 µmol/mL of cystine in 0.1 N HCl. Norleucine (N8513, Sigma, St. Louis, MO, USA) was used as the internal standard. To prepare the mixture, 80 µL of the amino acid standard was combined with 80 µL of norleucine solution (2.5 µmol/mL) and then dried. The mixture was derivatized with 100 µL of PITC solution for 30 min and freeze-dried. The residue was dissolved in 1 mL of buffer and filtered (0.2 µm) to obtain the stock solution, which was diluted to 100, 50, and 25 nmol/mL for calibration.

Analyses were conducted utilizing a Dionex Ultimate 3000 system (Thermo Scientific, Waltham, MA, USA), which featured a pump, autosampler, column compartment, and photodiode array detector. The system was operated via Chromeleon 6.8 software. Chromatographic separation was performed on a Waters Pico•Tag C18 column (4 µm, 3.9 × 150 mm, Waters Corporation) accompanied by a Nova-Pak guard column (4 µm, 3.9 × 20 mm, Waters Corporation) at a controlled temperature of 38 °C. The mobile phases consisted of eluent A (140 mM sodium acetate, 0.05% triethylamine, pH 6.40 adjusted with glacial acetic acid, supplemented with 60 mL/L acetonitrile) and eluent B (60% acetonitrile in water). Detection occurred at 254 nm, with samples injected in volumes of 20 µL.

The gel strength was determined by following Yang et al. [19]. Freeze-dried gelatin was dissolved at 6.67% (*w*/*w*) in distilled water, allowed to swell, then heated at 60 °C and stirred until fully dissolved. The liquid was placed in a vacuum pouch, sealed in a vacuum sealer (VacMaster^®^ VP545, Ary Inc. Kansas City, MO, USA), laid flat, and matured at 10 °C for 17 ± 1 h in a refrigerator (Environmental Growth Chambers). The gelatin sheet was removed from its vacuum-sealed packaging and sectioned into 1-inch cubes. Gel strength was evaluated using a TA XT Plus Texture Analyzer (Texture Technologies Corp., Hamilton, MA, USA) equipped with a 12.5 mm diameter flat plastic plunger. The plunger was driven 4 mm into the gelatin sample with a 5 kg load cell at a rate of 1 mm/s. The peak force measured in grams was recorded as the “bloom” strength at 10 °C [19]. Measurements were taken immediately after removing the samples from the refrigerator to ensure a temperature of 10 °C.

The gel melting point was determined by following the Ninan et al. [55] procedure. The gelatin solutions were prepared at 6.67% (*w*/*w*) with distilled water. Five ml aliquots of sample were transferred to 50 mL VWR centrifuge tubes (VWR International, LLC). Sample tubes were degassed in a vacuum chamber (VacMaster^®^ VP545, Ary Inc.). Tubes were sealed with parafilm, heated at 60 °C in a water bath for 15 min, then cooled in ice water. The gel matured at 10 °C in a refrigerator for 16–18 h. A mixture of chloroform (Macron Fine Chemicals™, Radnor, PA, USA) and methyl red (Fluka Analytical™, Charlotte, NC, USA) was placed on the surface of the gel immediately before analysis. Tubes were placed in a water bath starting at 10 °C, and the water was heated at a rate of 0.2 °C per minute using an immersion circulator (Anova Precision^®^ Cooker, San Francisco, CA, USA). The melting point was recorded as the temperature when colored drops started moving freely down the gel [55].

The gel-setting temperature was determined by following the Muyonga et al. [95] and Ninan et al. [55] procedures. The gelatin solutions were prepared at 10% (*w*/*w*) with distilled water. Five ml aliquots of the sample were transferred to 50 mL centrifuge tubes (VWR International, LLC). Sample tubes were degassed in a vacuum chamber (VacMaster^®^ VP545, Ary Inc.). The tubes were covered with parafilm (Bemis Flexible Packaging, Neenah, WI, USA) and heated in a water bath at 60 °C for 15 min. Test tubes containing dissolved samples were transferred to another water bath maintained at 10 °C. A thermometer was placed into the sample and removed every 30 s. The temperature at which the gelatin solution stopped dripping from the tip of the thermometer was noted as the setting temperature [55,95].

### 4.3. Evaluation of Potassium Sorbate, Lactic Acid, Gelatin, Gelatin with Potassium Sorbate, and Gelatin with Lactic Acid

#### 4.3.1. Catfish Fillet Collection and Sample Preparation

Fresh farm-raised Channel Catfish fillets (*Ictalurus punctatus*) (198 ± 2 g) were obtained from a local processor (Breaux Bridge, LA, USA) and transported to the Seafood Quality Laboratory at Louisiana State University (Baton Rouge, LA, USA). Samples were transported in ice chests with ice to maintain the temperature under 4 °C. Samples were prepared and treated within 12 h of collection. Catfish fillets were cut to a final weight of 100 ± 1 g. Samples were randomly assigned to the treatments (described below).

#### 4.3.2. Preparation of Antimicrobial Solution

Antimicrobial solutions were formulated immediately prior to the treatment of catfish fillets. This project comprised two experiments. In the first experiment, three solutions were utilized: potassium sorbate (PS) (VWR International, LLC) at 2% *w*/*w*, lactic acid (LA) (Chivine Resources Inc., Bloomfield, CT, USA) at 1% *w*/*w*, and a control (C) consisting of deionized water rinsing. The second experiment involved four solutions: gelatin (G), gelatin with potassium sorbate (G+PS), gelatin with lactic acid (G+LA), and a control (C) rinsed with deionized water. Gelatin treatments were prepared by creating a base solution, which was subsequently combined with either potassium sorbate (PS; 2% *w*/*w*) or lactic acid (LA; 1% *w*/*w*) [8,96]. The base solution consisted of 5% (*w*/*w*, dry weight basis) catfish skin gelatin dissolved in deionized water at 50 °C. Glycerol (VWR International, LLC) was then added at 20% (*w*/*w*) relative to the gelatin content, followed by incorporation of the antimicrobials (PS or LA). Glycerol was used as a plasticizer to improve coating flexibility, consistent with commonly reported levels for protein-based edible films [97,98].

#### 4.3.3. Catfish Fillets Treated with Antimicrobial Solutions

Catfish fillets were placed in sterile plastic bags (Whirl-Pak Poultry Rinse Bag, Filtration Group, Chicago, IL, USA) and exposed to each treatment for 60 s, using twice the volume of solution relative to the weight of the fish. This method ensured thorough contact between the fillets and the treatments. Then, fillets were removed from the treatment solutions and placed on stainless steel cooling racks (Hamilton Housewares, Amazon, Seattle, WA, USA) for 10 min at room temperature (21.7 °C) for draining and air-drying. After drying, all the fillets treated with the three gelatin preparations had a thin layer of gelatin that was visible on the surface of the fillets. Each fillet was placed in an individual plastic bag (Ziploc^®^, S.C. Johnson & Son Inc., Racine, WI, USA) and stored in ice at 0.5 ± 2 °C in a walk-in refrigerated unit (Environmental Growth Chambers) to simulate industry-standard iced storage conditions used during seafood handling, distribution, and retail. This approach reflects common practices designed to maintain fish as close as possible to 0 °C, reducing microbial and enzymatic activity. Although retail refrigeration may occur at slightly higher temperatures (2–4 °C), iced storage was selected to represent optimal cold-chain conditions [4]. For each treatment, two replicates (fillets) were prepared for each storage day. Samples were analyzed on day 0 and at 3-day intervals up to day 18 for the first experiment, and up to day 30 for the second experiment. Ice was replenished every day of analysis. The study involved an analysis of physical, chemical, and microbial activities.

### 4.4. Microbial Analyses

Microbial analyses were performed for Aerobic Plate Count (APC), Psychrophilic, *Pseudomonas* spp., and yeasts after the treatments. Twenty-five g of fish was homogenized with 25 mL of phosphate-buffered saline (PBS), followed by serial dilutions, and then plated in duplicate. Analyses for APC and Psychrophilic were performed using Standard Method Agar (Neogen, Lasing, MI, USA) incubating the samples at 35 ± 2 °C for 48 h and 10 ± 2 °C for 10 days, respectively [99]. *Pseudomonas* spp. was analyzed using *Pseudomonas* agar base with CFC Supplement (FD036-5X5VL) (Himedia, Mumbai, India) incubated at 25 ± 2 °C for 48 h [100]. Petrifilm™ Rapid Yeasts and Mold Count Plates from 3M™ (3M™ Microbiology, St. Paul, MN, USA) were used for yeasts and were incubated at 25 ± 2 °C for 72 h [99].

### 4.5. Physical and Chemical Analyses

Physical analyses included color and moisture content. Color parameters were measured using Hunter color scale values (L, a*, and b*) with a Baking meter BC-10 colorimeter (Konica Minolta Business Solutions USA, Inc., Ramsey, NJ, USA); the CR-A43 calibration plate was used for calibration (Konica Minolta). The L measures the lightness on a 0–100 scale (white being 100 and black 0), the a* measures redness (red being a positive value and green a negative value), and the b* measures yellowness (yellow being a positive value and blue a negative value). Previous studies have measured color randomly on one or both sides of the fillet; however, none reported any differences between the sides [14,33,91]. A preliminary trial identified a statistically significant difference between the skin side and the bone side (*p* < 0.05). Consequently, color measurements were taken on the skin side in three locations: the head end, middle, and tail end.

Moisture analysis was performed employing the standard oven-drying technique. Precisely three grams of processed catfish sample were measured into aluminum weighing dishes (Heathrow Scientific SE, Vernon Hills, IL, USA) and subsequently placed in an oven maintained at 105 °C (Model 658, ThermoFisher Scientific) for 24 h to facilitate complete moisture removal. The moisture content was determined by calculating the difference in sample weight before and after drying.

Chemical analyses were conducted for thiobarbituric acid reactive substances (TBARS) and pH. Thiobarbituric acid reactive substances analysis was conducted following the Lemon [101] method by measuring the amount of malondialdehyde (MDA) in the fillets and reporting as thiobarbituric acid (TBA) value in units of mg MDA per kg of fillet. Optical density (OD) was measured at 532 nm using a spectrophotometer-Spectronic 200 (ThermoFisher Scientific). pH was measured using a pH/mV Meter SX811-SS with a LabSen753 spear pH (Apera Instruments, Columbus, OH, USA), designed for solid and semi-solid food samples. pH was measured directly from the fish flesh in three random spots in the catfish fillet.

### 4.6. Statistical Analysis

For catfish skin gelatin characterization, six independent replicates were analyzed, and results are expressed as mean ± standard deviation. Shelf-life studies were conducted in two independent trials for each treatment, with all analytical measurements performed in duplicate. Data are presented as mean ± standard deviation.

Statistical analyses were performed using JMP Statistical Discovery software (version 15 Pro; SAS Institute Inc., Cary, NC, USA). Analysis of variance (ANOVA) was applied to evaluate the effects of treatment and storage time. When significant differences were detected, mean comparisons were conducted using Tukey’s test at a significance level of α = 0.05. Comparisons were performed both among treatments within each storage day and across storage days within each treatment.

## Figures and Tables

**Table 1:** Comparison of amino acid values (number of residues/1000) found in skin gelatin from Channel Catfish (*Ictalurus punctatus*) mean ± standard deviation (*n* = 6) compared to African Catfish (*Clarias gariepinus*), Tilapia (*Oreochromis niloticus*), Megrim (*Lepidorhombus whiffiagonis*), and Cod (*Gadus morhua*) [24,44,45].

Amino Acid	Catfish (*Ictalurus punctatus*)	African Catfish (*Clarias* *gariepinus*)	Tilapia (*Oreochromis niloticus*)	Megrim (*Lepidorhombus whiffiagonis*)	Cod (*Gadus morhua*)
Aspartic acid and asparagine	43 ± 0.71	50	48	48	52
Glutamic acid and glutamine	75 ± 2.12	85	69	72	78
Serine	41 ± 1.41	38	35	41	64
Glycine	392 ± 0.71	250	347	350	344
Histidine	2 ± 0.00	11	6	8	8
Arginine	55 ± 0.71	78	47	54	56
Threonine	20 ± 0.00	25	24	20	25
Alanine	129 ± 1.41	86	123	123	96
Proline	113 ± 1.41	115	119	115	106
Tyrosine	4 ± 0.00	6	2	3	3
Valine	24 ± 0.71	22	15	18	18
Methionine	11 ± 1.41	92	9	13	17
Isoleucine	12 ± 0.71	13	8	8	11
Leucine	20 ± 0.71	22	23	21	22
Phenylalanine	41 ± 2.83	16	13	14	16
Lysine	22 ± 2.12	33	25	27	29

## Data Availability

The original contributions presented in this study are included in the article/Supplementary Materials. Further inquiries can be directed to the corresponding author.

## Supplementary Materials

The following supporting information can be downloaded at: https://www.mdpi.com/article/10.3390/gels12070584/s1, Tables S1 and S2: Aerobic Plate Counts (APC); Tables S3 and S4: Psychrophilic counts; Tables S5 and S6: *Pseudomonas* spp.; Tables S7 and S8: Yeasts counts; Tables S9 and S10: Moisture percentage; Tables S11 and S12: pH values; Tables S13 and S14: L* colorimeter values; Tables S15 and S16: a* colorimeter values; Tables S17 and S18: b* colorimeter values.

## Abbreviations

The following abbreviations are used in this manuscript:

ANOVA	Analysis of variance
APC	Aerobic plate count
CFU	Colony-forming units
TBARS	Thiobarbituric acid reactive substances
MDA	Malondialdehyde
PBS	Phosphate-buffered saline
L*	Lightness
a*	Red/green coordinate
b*	Yellow/blue coordinate
C	Control
G	Gelatin
LA	Lactic acid
PS	Potassium sorbate
G+LA	Gelatin + lactic acid
G+PS	Gelatin + potassium sorbate
GMP	Good manufacturing practice
ICMSF	International Commission on Microbiological Specifications for Foods
