# Supplementary material for: Evaluation of Catfish Skin Gelatin-Based Edible Antimicrobial Coating with Lactic Acid and Potassium Sorbate on the Shelf Life and Quality of Fresh Catfish Fillets
Source: Gels. 2026 Jul 2;12(7):584. doi: 10.3390/gels12070584 (PMC13409432; doi:10.3390/gels12070584)
Supplement: Supplementary file 1 [file gels-12-00584-s001.zip › Table S1 and S2 Aerobic plate count.pdf]

Table S1. Aerobic Plate Counts (APC) during18-day shelf life study of catfish fillets comparing antimicrobial coatings: untreated (C), Potassium sorbate (PS), and Lactic acid (LA). Log CFU/g: Logarithmic Colony Forming Units per gram of sample. Mean ± Standard Deviation values within each row with different capital letters indicate treatments are significantly different within each day of storage ($p < 0.05$), while different lowercase letters within each column indicate days of storage are significantly different within each individual treatment ($p < 0.05$).

**Day**	**C**					**LA**					**PS**				
0	3.72	±	0.17	b	A	3.02	±	0.19	a	B	3.43	±	0.12	a	AB
3	3.73	±	0.13	b	A	2.66	±	0.04	a	B	3.54	±	0.01	a	A
6	3.64	±	0.26	b	A	2.92	±	0.50	a	A	3.90	±	0.89	a	A
9	3.88	±	0.22	ab	A	3.66	±	0.28	a	A	3.37	±	0.19	a	A
12	4.75	±	0.64	ab	A	3.45	±	0.52	a	A	3.36	±	0.05	a	A
15	5.33	±	0.61	a	A	3.14	±	0.05	a	B	3.35	±	0.00	a	B
18	3.74	±	0.12	b	A	3.97	±	0.94	a	A	3.26	±	0.02	a	A

Table S2. Aerobic Plate Counts (APC) during 30-day shelf-life study of catfish fillets comparing antimicrobial coatings: untreated (C), Gelatin (G), Gelatin + Lactic acid (G+LA), and Gelatin + Potassium sorbate (G+PS). Log CFU/g: Logarithmic Colony Forming Units per gram of sample. Mean ± Standard Deviation values within each row with different capital letters indicate treatments are significantly different within each day of storage ($p < 0.05$), while different lowercase letters within each column indicate days of storage are significantly different within each individual treatment ($p < 0.05$).

**Day**	**C**					**G**					**G+LA**					**G+PS**				
0	4.50	±	0.21	e	A	4.08	±	0.10	f	A	3.55	±	0.03	d	B	4.20	±	0.01	a	A
3	4.31	±	0.13	ef	A	4.05	±	0.19	f	AB	3.59	±	0.01	d	B	3.99	±	0.07	a	AB
6	3.87	±	0.24	f	A	4.19	±	0.45	ef	A	3.23	±	0.24	d	A	3.76	±	0.72	a	A
9	4.47	±	0.12	ef	A	4.21	±	0.07	ef	AB	3.29	±	0.04	d	C	4.03	±	0.11	a	B
12	5.55	±	0.05	d	A	4.83	±	0.05	de	B	3.68	±	0.26	d	C	3.66	±	0.09	a	C
15	6.08	±	0.09	cd	A	5.51	±	0.20	cd	A	3.25	±	0.15	d	B	3.77	±	0.11	a	B
18	6.48	±	0.17	c	A	5.60	±	0.11	c	AB	4.69	±	0.15	bc	BC	3.95	±	0.38	a	C
21	6.57	±	0.13	bc	A	6.82	±	0.04	b	A	4.02	±	0.33	cd	B	4.13	±	0.01	a	B
24	7.36	±	0.07	a	A	7.20	±	0.12	ab	A	5.40	±	0.33	ab	B	4.25	±	0.25	a	C
27	7.42	±	0.24	a	A	7.29	±	0.13	ab	A	5.92	±	0.11	a	B	4.40	±	0.25	a	C
30	7.17	±	0.10	ab	A	7.85	±	0.03	a	A	5.77	±	0.28	a	B	4.40	±	0.34	a	C
