# Supplementary material for: Evaluation of Catfish Skin Gelatin-Based Edible Antimicrobial Coating with Lactic Acid and Potassium Sorbate on the Shelf Life and Quality of Fresh Catfish Fillets
Source: Gels. 2026 Jul 2;12(7):584. doi: 10.3390/gels12070584 (PMC13409432; doi:10.3390/gels12070584)
Supplement: Supplementary file 1 [file gels-12-00584-s001.zip › Table S11 and S12 pH values.pdf]

Table S11. pH values 18-day shelf-life study of catfish fillets comparing antimicrobial coatings: untreated (C), Potassium sorbate (PS), and Lactic acid (LA). Mean ± Standard Deviation values within each row with different capital letters indicate treatments are significantly different within each day of storage ($p < 0.05$), while different lowercase letters within each column indicate days of storage are significantly different within each individual treatment ($p < 0.05$).

**Day**	**C**					**LA**					**PS**				
0	6.87	±	0.10	a	A	6.85	±	0.13	a	A	6.68	±	0.14	b	B
3	6.72	±	0.12	ab	A	6.73	±	0.21	ab	A	6.68	±	0.09	b	A
6	6.76	±	0.11	ab	B	6.80	±	0.13	ab	B	7.00	±	0.11	a	A
9	6.69	±	0.07	ab	A	6.43	±	0.29	bc	B	6.65	±	0.07	b	A
12	6.74	±	0.17	ab	AB	6.52	±	0.29	abc	B	7.00	±	0.05	a	A
15	6.63	±	0.10	b	A	6.28	±	0.20	cd	B	6.69	±	0.12	b	A
18	6.61	±	0.11	b	B	5.99	±	0.20	d	C	6.97	±	0.20	a	A

Table S12. pH values 30-day shelf-life study of catfish fillets comparing antimicrobial coatings: untreated (C), Gelatin (G), Gelatin + Lactic acid (G+LA), and Gelatin + Potassium sorbate (G+PS). Mean ± Standard Deviation values within each row with different capital letters indicate treatments are significantly different within each day of storage ($p < 0.05$), while different lowercase letters within each column indicate days of storage are significantly different within each individual treatment ($p < 0.05$).

**Day**	**C**					**G**					**G+LA**					**G+PS**				
0	6.69	±	0.24	ab	C	6.72	±	0.08	a	BC	6.95	±	0.11	a	AB	6.98	±	0.12	a	A
3	6.73	±	0.06	ab	A	6.62	±	0.06	ab	AB	6.40	±	0.20	bcd	B	6.56	±	0.18	bcd	AB
6	6.67	±	0.04	ab	A	6.71	±	0.06	a	A	6.65	±	0.18	abc	A	6.71	±	0.12	b	A
9	6.66	±	0.09	ab	AB	6.71	±	0.17	a	A	6.70	±	0.14	ab	A	6.47	±	0.06	cd	B
12	6.76	±	0.17	a	A	6.66	±	0.08	ab	AB	6.44	±	0.17	bcd	B	6.68	±	0.21	bc	AB
15	6.57	±	0.08	abc	AB	6.63	±	0.09	ab	A	6.40	±	0.14	bcd	B	6.73	±	0.12	b	A
18	6.58	±	0.09	abc	AB	6.52	±	0.14	bc	AB	6.42	±	0.12	bcd	B	6.66	±	0.06	bc	A
21	6.69	±	0.08	ab	A	6.67	±	0.05	ab	A	6.38	±	0.19	cd	B	6.64	±	0.08	bcd	A
24	6.37	±	0.10	cd	B	6.62	±	0.03	ab	A	6.52	±	0.14	bcd	AB	6.63	±	0.08	bcd	A
27	6.52	±	0.13	bc	AB	6.60	±	0.11	ab	A	6.33	±	0.10	d	C	6.43	±	0.04	d	BC
30	6.21	±	0.13	d	C	6.35	±	0.07	c	BC	6.43	±	0.18	bcd	B	6.64	±	0.10	bcd	A
