# Supplementary material for: Evaluation of Catfish Skin Gelatin-Based Edible Antimicrobial Coating with Lactic Acid and Potassium Sorbate on the Shelf Life and Quality of Fresh Catfish Fillets
Source: Gels. 2026 Jul 2;12(7):584. doi: 10.3390/gels12070584 (PMC13409432; doi:10.3390/gels12070584)
Supplement: Supplementary file 1 [file gels-12-00584-s001.zip › Table S13 and S14 L colorimeter values.pdf]

Table S13. L* colorimeter values during 18-day shelf-life study of catfish fillets comparing antimicrobial coatings: untreated (C), Potassium sorbate (PS), and Lactic acid (LA). Mean ± Standard Deviation values within each row with different capital letters indicate treatments are significantly different within each day of storage ($p < 0.05$), while different lowercase letters within each column indicate days of storage are significantly different within each individual treatment ($p < 0.05$).

Day	C					LA					PS				
0	65.7	±	2.5	a	A	62.5	±	3.3	b	A	62.6	±	3.2	ab	A
3	61.9	±	1.8	a	A	63.3	±	2.5	ab	A	61.4	±	3.1	ab	A
6	61.6	±	4.1	a	A	66.8	±	1.3	a	A	66.9	±	7.3	a	A
9	62.9	±	2.7	a	A	65.3	±	2.2	ab	A	63.4	±	2.2	ab	A
12	62.8	±	4.3	a	AB	66.6	±	0.9	ab	A	61.2	±	2.8	ab	B
15	63.1	±	3.3	a	AB	65.5	±	2.1	ab	A	60.8	±	2.2	ab	B
18	62.8	±	3.8	a	AB	63.8	±	2.6	ab	A	59.3	±	1.3	b	B

Table S14. L* colorimeter values during 30-day shelf-life study of catfish fillets comparing antimicrobial coatings: untreated (C), Gelatin (G), Gelatin + Lactic acid (G+LA), and Gelatin + Potassium sorbate (G+PS). Mean ± Standard Deviation values within each row with different capital letters indicate treatments are significantly different within each day of storage ($p < 0.05$), while different lowercase letters within each column indicate days of storage are significantly different within each individual treatment ($p < 0.05$).

Day	C					G					G+LA					G+PS				
0	63.9	±	1.1	b	A	64.6	±	1.4	a	A	64.4	±	0.4	bc	A	65.8	±	1.3	a	A
3	66.0	±	0.8	ab	A	66.1	±	1.5	a	A	70.2	±	5.9	a	A	66.3	±	1.2	a	A
6	64.0	±	2.1	b	A	66.5	±	4.1	a	A	67.7	±	1.2	abc	A	64.6	±	3.9	a	A
9	67.8	±	2.0	a	A	67.0	±	2.2	a	A	66.8	±	0.9	abc	A	66.2	±	1.9	a	A
12	66.4	±	1.6	ab	A	67.0	±	1.5	a	A	67.1	±	1.8	abc	A	65.2	±	1.2	a	A
15	67.3	±	2.3	ab	A	65.5	±	1.4	a	AB	63.7	±	1.4	c	B	65.6	±	1.9	a	AB
18	66.6	±	2.0	ab	A	67.1	±	2.5	a	A	66.1	±	2.4	abc	A	67.6	±	1.3	a	A
21	65.8	±	1.8	ab	A	65.5	±	2.4	a	A	68.1	±	0.9	ab	A	66.9	±	1.2	a	A
24	65.6	±	2.3	ab	A	65.8	±	2.6	a	A	67.0	±	1.5	abc	A	68.0	±	4.3	a	A
27	67.3	±	1.5	ab	A	64.2	±	1.4	a	B	66.4	±	1.5	abc	AB	65.6	±	1.2	a	AB
30	67.1	±	1.2	ab	A	66.6	±	2.6	a	A	65.7	±	1.4	bc	A	64.5	±	1.7	a	A
