# Supplementary material for: Evaluation of Catfish Skin Gelatin-Based Edible Antimicrobial Coating with Lactic Acid and Potassium Sorbate on the Shelf Life and Quality of Fresh Catfish Fillets
Source: Gels. 2026 Jul 2;12(7):584. doi: 10.3390/gels12070584 (PMC13409432; doi:10.3390/gels12070584)
Supplement: Supplementary file 1 [file gels-12-00584-s001.zip › Table S15 and S16 a colorimeter values.pdf]

Table S15. a* colorimeter values during 18-day shelf-life study of catfish fillets comparing antimicrobial coatings: untreated (C), Potassium sorbate (PS), and Lactic acid (LA). Mean ± Standard Deviation values within each row with different capital letters indicate treatments are significantly different within each day of storage ($p < 0.05$), while different lowercase letters within each column indicate days of storage are significantly different within each individual treatment ($p < 0.05$).

Day			C					LA					PS		
0	-0.2	±	1.6	a	A	1.6	±	3.0	a	A	0.7	±	1.8	a	A
3	1.1	±	1.6	a	A	1.5	±	2.6	a	A	1.4	±	3.1	a	A
6	1.6	±	2.5	a	A	0.1	±	2.0	a	A	0.3	±	1.9	a	A
9	2.2	±	2.8	a	A	0.5	±	2.1	a	A	1.1	±	2.8	a	A
12	0.9	±	3.1	a	A	0.8	±	1.1	a	A	2.4	±	1.8	a	A
15	1.3	±	2.2	a	A	-0.2	±	1.8	a	A	1.9	±	2.3	a	A
18	2.4	±	2.6	a	A	1.3	±	2.6	a	A	1.6	±	2.1	a	A

Table S16. a* colorimeter values during 30-day shelf-life study of catfish fillets comparing antimicrobial coatings: untreated (C), Gelatin (G), Gelatin + Lactic acid (G+LA), and Gelatin + Potassium sorbate (G+PS). Mean ± Standard Deviation values within each row with different capital letters indicate treatments are significantly different within each day of storage ($p < 0.05$), while different lowercase letters within each column indicate days of storage are significantly different within each individual treatment ($p < 0.05$).

Day			C					G					LA					PS		
0	0.67	±	0.53	a	A	0.28	±	1.22	a	AB	-0.67	±	0.82	a	AB	-1.13	±	1.26	a	B
3	-0.48	±	2.24	a	A	0.13	±	2.18	a	A	0.38	±	2.26	a	A	-0.22	±	1.92	a	A
6	0.52	±	2.07	a	A	0.70	±	2.57	a	A	-0.02	±	1.18	a	A	-0.42	±	3.38	a	A
9	-0.22	±	0.71	a	A	0.37	±	1.62	a	A	-0.37	±	1.26	a	A	0.72	±	2.20	a	A
12	0.92	±	1.30	a	A	0.70	±	1.45	a	A	0.95	±	1.68	a	A	1.07	±	0.89	a	A
15	0.95	±	1.82	a	A	-0.43	±	1.27	a	A	1.67	±	1.51	a	A	0.47	±	2.60	a	A
18	1.05	±	2.18	a	A	0.97	±	1.34	a	A	0.93	±	0.92	a	A	-0.32	±	0.92	a	A
21	0.25	±	1.16	a	A	0.35	±	1.06	a	A	0.07	±	1.30	a	A	0.12	±	0.83	a	A
24	0.92	±	1.61	a	A	1.08	±	1.72	a	A	0.33	±	1.21	a	A	-0.65	±	0.86	a	A
27	0.08	±	1.17	a	AB	1.43	±	1.79	a	A	-0.27	±	0.63	a	AB	-0.65	±	0.74	a	B
30	0.17	±	2.19	a	A	0.27	±	1.31	a	A	0.28	±	1.44	a	A	1.12	±	1.66	a	A
