# Supplementary material for: Evaluation of Catfish Skin Gelatin-Based Edible Antimicrobial Coating with Lactic Acid and Potassium Sorbate on the Shelf Life and Quality of Fresh Catfish Fillets
Source: Gels. 2026 Jul 2;12(7):584. doi: 10.3390/gels12070584 (PMC13409432; doi:10.3390/gels12070584)
Supplement: Supplementary file 1 [file gels-12-00584-s001.zip › Table S17 and S18 b colorimeter values.pdf]

Table S17. b* colorimeter values during 18-day shelf-life study of catfish fillets comparing antimicrobial coatings: untreated (C), Lactic acid (LA), and Potassium sorbate (PS). Mean ± Standard Deviation values within each row with different capital letters indicate treatments are significantly different within each day of storage ($p < 0.05$), while different lowercase letters within each column indicate days of storage are significantly different within each individual treatment ($p < 0.05$).

Day	C					LA					PS				
0	1.9	±	2.2	b	B	3.6	±	2.0	b	A	2.0	±	1.6	b	B
3	4.5	±	2.8	ab	B	5.6	±	2.6	ab	A	3.0	±	2.5	ab	B
6	3.7	±	2.5	b	B	2.7	±	2.2	b	A	1.3	±	2.2	b	B
9	3.3	±	3.1	ab	B	6.6	±	3.1	ab	A	2.7	±	1.8	ab	B
12	3.5	±	2.6	ab	B	6.5	±	2.0	ab	A	4.6	±	2.6	ab	B
15	3.1	±	2.3	ab	B	6.4	±	3.5	ab	A	2.8	±	1.8	ab	B
18	3.7	±	3.0	a	B	7.5	±	3.5	a	A	4.6	±	2.4	a	B

Table S18. b* colorimeter values during 30-day shelf-life study of catfish fillets comparing antimicrobial coatings: untreated (C), Gelatin (G), Gelatin + Lactic acid (G+LA), and Gelatin + Potassium sorbate (G+PS). Mean ± Standard Deviation values within each row with different capital letters indicate treatments are significantly different within each day of storage ($p < 0.05$), while different lowercase letters within each column indicate days of storage are significantly different within each individual treatment ($p < 0.05$).

Day	C					G					G+LA					G+PS				
0	1.62	±	0.71	a	A	1.20	±	0.96	a	A	1.52	±	1.14	a	A	0.25	±	1.55	a	A
3	1.05	±	1.84	a	A	0.95	±	2.39	a	A	1.97	±	4.02	a	A	1.05	±	1.44	a	A
6	1.58	±	1.29	a	A	2.77	±	1.06	a	A	3.23	±	0.91	a	A	1.25	±	1.80	a	A
9	1.93	±	0.76	a	A	1.35	±	1.16	a	A	1.80	±	1.18	a	A	1.88	±	1.35	a	A
12	1.65	±	0.52	a	B	1.53	±	0.89	a	B	3.67	±	1.01	a	A	2.05	±	1.14	a	B
15	2.07	±	0.76	aa	AB	0.92	±	0.62	a	B	3.95	±	1.26	a	A	1.08	±	1.85	a	AB
18	1.98	±	0.79	a	B	3.10	±	0.67	a	AB	3.58	±	1.49	a	A	2.00	±	0.65	a	B
21	1.85	±	0.79	a	B	1.97	±	0.74	a	B	3.65	±	1.14	a	A	1.58	±	0.63	a	B
24	1.95	±	0.37	a	B	2.35	±	1.36	a	B	4.47	±	1.32	a	A	0.72	±	0.64	a	B
27	2.32	±	0.40	a	A	2.38	±	1.54	a	A	2.10	±	1.00	a	A	1.40	±	1.40	a	A
30	2.73	±	1.04	a	A	2.42	±	0.91	a	A	3.12	±	1.77	a	A	1.63	±	1.53	a	A
