# Supplementary material for: Evaluation of Catfish Skin Gelatin-Based Edible Antimicrobial Coating with Lactic Acid and Potassium Sorbate on the Shelf Life and Quality of Fresh Catfish Fillets
Source: Gels. 2026 Jul 2;12(7):584. doi: 10.3390/gels12070584 (PMC13409432; doi:10.3390/gels12070584)
Supplement: Supplementary file 1 [file gels-12-00584-s001.zip › Table S3 and S4 Psychrophilic count.pdf]

Table S3. Psychrophilic counts during 18-day shelf-life study of catfish fillets comparing antimicrobial coatings: untreated (C), Potassium sorbate (PS), and Lactic acid (LA). Log CFU/g: Logarithmic Colony Forming Units per gram of sample. Mean ± Standard Deviation values within each row with different capital letters indicate treatments are significantly different within each day of storage ($p < 0.05$), while different lowercase letters within each column indicate days of storage are significantly different within each individual treatment ($p < 0.05$).

Day	C					LA					PS				
0	3.72	±	0.17	b	A	3.02	±	0.19	a	A	3.43	±	0.12	a	A
3	3.73	±	0.13	b	A	2.66	±	0.04	a	B	3.54	±	0.01	a	A
6	3.64	±	0.26	b	A	2.92	±	0.50	a	A	3.90	±	0.89	a	A
9	3.88	±	0.22	ab	A	3.66	±	0.28	a	A	3.37	±	0.19	a	A
12	4.75	±	0.64	ab	A	3.45	±	0.52	a	A	3.36	±	0.05	a	A
15	5.33	±	0.61	a	A	3.14	±	0.05	a	B	3.35	±	0.00	a	B
18	3.74	±	0.12	b	A	3.97	±	0.94	a	A	3.26	±	0.02	a	A

Table S4. Psychrophilic counts during 30-day shelf-life study of catfish fillets comparing antimicrobial coatings: untreated (C), Gelatin (G), Gelatin + Lactic acid (G+LA), and Gelatin + Potassium sorbate (G+PS). Log CFU/g: Logarithmic Colony Forming Units per gram of sample. Mean ± Standard Deviation values within each row with different capital letters indicate treatments are significantly different within each day of storage ($p < 0.05$), while different lowercase letters within each column indicate days of storage are significantly different within each individual treatment ($p < 0.05$).

Day	C					G					G+LA					G+PS				
0	4.67	±	0.42	e	A	4.11	±	0.10	e	A	3.77	±	0.14	e	B	3.12	±	0.01	f	AB
3	4.36	±	0.04	e	A	4.20	±	0.22	e	AB	3.54	±	0.02	e	C	3.28	±	0.28	f	BC
6	4.21	±	0.07	e	A	4.43	±	0.22	e	A	3.99	±	0.26	e	A	3.75	±	0.24	f	A
9	6.01	±	0.34	d	A	6.00	±	0.06	d	A	5.39	±	0.07	d	A	5.49	±	0.20	e	A
12	7.46	±	0.02	c	A	7.52	±	0.05	c	A	6.67	±	0.13	cd	B	6.00	±	0.40	d	AB
15	8.36	±	0.03	b	A	8.00	±	0.64	bc	AB	7.45	±	0.05	c	B	6.82	±	0.18	c	AB
18	8.72	±	0.14	ab	A	8.59	±	0.11	ab	A	8.20	±	0.07	b	B	7.86	±	0.22	b	AB
21	8.91	±	0.16	ab	AB	9.05	±	0.18	a	A	8.60	±	0.07	ab	B	8.26	±	0.21	ab	AB
24	8.94	±	0.02	ab	A	9.29	±	0.01	a	A	8.47	±	0.16	ab	B	8.10	±	0.09	ab	B
27	9.06	±	0.09	ab	A	9.30	±	0.02	a	A	8.66	±	0.29	ab	A	8.70	±	0.34	ab	A
30	9.18	±	0.06	a	A	9.24	±	0.05	a	A	8.91	±	0.21	a	A	8.89	±	0.13	a	A
