# Supplementary material for: Evaluation of Catfish Skin Gelatin-Based Edible Antimicrobial Coating with Lactic Acid and Potassium Sorbate on the Shelf Life and Quality of Fresh Catfish Fillets
Source: Gels. 2026 Jul 2;12(7):584. doi: 10.3390/gels12070584 (PMC13409432; doi:10.3390/gels12070584)
Supplement: Supplementary file 1 [file gels-12-00584-s001.zip › Table S5 and S6 Pseudomonas counts.pdf]

Table S5. *Pseudomonas* spp. counts during 18-day shelf-life study of catfish fillets comparing antimicrobial coatings: untreated (C), Lactic acid (LA), and Potassium sorbate (PS). Log CFU/g: Logarithmic Colony Forming Units per gram of sample. Mean ± Standard Deviation values within each row with different capital letters indicate treatments are significantly different within each day of storage ($p < 0.05$), while different lowercase letters within each column indicate days of storage are significantly different within each individual treatment ($p < 0.05$).

**Day**			**C**					**LA**					**PS**		
0	3.26	±	0.17	c	A	3.15	±	0.05	d	A	2.84	±	0.16	c	A
3	3.34	±	0.12	c	A	2.84	±	0.02	d	B	3.32	±	0.00	c	A
6	3.93	±	0.09	c	A	3.03	±	0.11	d	B	3.60	±	0.18	c	A
9	5.10	±	0.30	b	A	4.29	±	0.11	c	A	4.95	±	0.41	b	A
12	7.30	±	0.03	a	A	6.07	±	0.10	b	B	7.05	±	0.11	a	A
15	7.67	±	0.47	a	A	7.30	±	0.20	a	A	7.58	±	0.53	a	A
18	7.62	±	0.11	a	A	6.92	±	0.25	a	A	7.58	±	0.18	a	A

Table S6. *Pseudomonas* spp. counts during 30-day shelf-life study of catfish fillets comparing antimicrobial coatings: untreated (C), Gelatin (G), Gelatin + Lactic acid (G+LA), and Gelatin + Potassium sorbate (G+PS). Log CFU/g: Logarithmic Colony Forming Units per gram of sample. Mean ± Standard Deviation values within each row with different capital letters indicate treatments are significantly different within each day of storage ($p < 0.05$), while different lowercase letters within each column indicate days of storage are significantly different within each individual treatment ($p < 0.05$).

**Day**			**C**					**G**					**G+LA**					**G+PS**		
0	2.42	±	0.06	e	AB	2.81	±	0.05	e	A	2.65	±	0.18	fg	A	2.20	±	0.08	e	B
3	2.78	±	0.11	e	A	2.81	±	0.18	e	A	1.75	±	0.21	g	B	2.23	±	0.12	e	AB
6	3.88	±	0.06	d	A	3.14	±	0.22	e	A	3.40	±	0.22	ef	A	3.00	±	0.38	de	A
9	4.83	±	0.26	c	A	4.55	±	0.08	d	AB	3.91	±	0.16	de	B	3.90	±	0.11	d	B
12	7.16	±	0.13	b	A	7.01	±	0.11	c	A	4.40	±	0.46	d	B	5.21	±	0.13	c	B
15	7.73	±	0.21	a	A	8.04	±	0.07	b	A	6.48	±	0.12	c	C	7.16	±	0.06	b	B
18	8.12	±	0.05	a	A	8.23	±	0.11	b	A	7.26	±	0.16	bc	A	7.74	±	0.51	ab	A
21	7.83	±	0.18	a	A	8.36	±	0.12	ab	A	7.82	±	0.38	ab	A	8.25	±	0.07	a	A
24	7.92	±	0.03	a	B	8.79	±	0.13	a	A	7.90	±	0.06	ab	B	7.90	±	0.21	ab	B
27	7.73	±	0.01	a	B	8.45	±	0.01	ab	A	8.17	±	0.21	ab	AB	8.08	±	0.13	ab	AB
30	8.08	±	0.16	a	A	8.38	±	0.03	ab	A	8.64	±	0.28	a	A	8.59	±	0.35	a	A
