# Supplementary material for: Evaluation of Catfish Skin Gelatin-Based Edible Antimicrobial Coating with Lactic Acid and Potassium Sorbate on the Shelf Life and Quality of Fresh Catfish Fillets
Source: Gels. 2026 Jul 2;12(7):584. doi: 10.3390/gels12070584 (PMC13409432; doi:10.3390/gels12070584)
Supplement: Supplementary file 1 [file gels-12-00584-s001.zip › Table S7 and S8 Yeast counts.pdf]

Table S7. Yeast counts during 18-day shelf-life study of catfish fillets comparing antimicrobial coatings: untreated (C), Lactic acid (LA), and Potassium sorbate (PS). Log CFU/g: Logarithmic Colony Forming Units per gram of sample. Mean ± Standard Deviation values within each row with different capital letters indicate treatments are significantly different within each day of storage ($p < 0.05$), while different lowercase letters within each column indicate days of storage are significantly different within each individual treatment ($p < 0.05$).

Day	C					LA					PS				
0	2.94	±	0.11	d	A	2.73	±	0.55	b	A	2.63	±	0.20	d	A
3	2.95	±	0.08	d	A	2.79	±	0.15	b	A	2.85	±	0.03	cd	A
6	3.29	±	0.04	d	A	3.17	±	0.35	b	A	3.07	±	0.04	cd	A
9	4.09	±	0.11	c	A	3.50	±	0.02	b	A	3.93	±	0.62	bc	A
12	4.78	±	0.19	b	A	5.51	±	0.29	a	A	4.90	±	0.47	ab	A
15	5.28	±	0.11	a	A	5.21	±	0.30	a	A	5.62	±	0.20	a	A
18	5.63	±	0.06	a	A	5.46	±	0.52	a	A	5.84	±	0.17	a	A

Table S8. Yeast counts during 30-day shelf-life study of catfish fillets comparing antimicrobial coatings: untreated (C), Gelatin (G), Gelatin + Lactic acid (G+LA), and Gelatin + Potassium sorbate (G+PS). Log CFU/g: Logarithmic Colony Forming Units per gram of sample. Mean ± Standard Deviation values within each row with different capital letters indicate treatments are significantly different within each day of storage ($p < 0.05$), while different lowercase letters within each column indicate days of storage are significantly different within each individual treatment ($p < 0.05$).

Day	C					G					G+LA					G+PS				
0	2.72	±	0.14	f	A	2.10	±	0.25	d	A	2.28	±	0.07	d	A	2.45	±	0.01	ef	A
3	2.89	±	0.05	ef	A	2.69	±	0.32	cd	A	2.33	±	0.06	d	A	2.19	±	0.25	ef	A
6	3.28	±	0.07	ef	A	2.74	±	0.15	cd	B	2.44	±	0.00	d	C	2.05	±	0.11	f	BC
9	3.63	±	0.18	de	A	3.26	±	0.22	c	A	3.46	±	0.36	c	A	3.00	±	0.09	e	A
12	4.32	±	0.09	cd	A	4.69	±	0.18	b	A	3.77	±	0.32	c	A	4.39	±	0.37	d	A
15	5.03	±	0.16	bc	A	4.89	±	0.31	ab	A	4.32	±	0.36	b	A	4.94	±	0.21	cd	A
18	5.83	±	0.17	a	A	5.20	±	0.04	ab	AB	5.50	±	0.12	ab	AB	5.11	±	0.26	bcd	B
21	5.27	±	0.50	ab	A	5.61	±	0.33	ab	A	6.07	±	0.38	ab	A	5.62	±	0.01	abc	A
24	5.64	±	0.07	ab	A	5.76	±	0.39	a	A	6.05	±	0.21	ab	A	5.86	±	0.38	ab	A
27	5.99	±	0.16	a	B	5.67	±	0.03	ab	BC	6.37	±	0.04	ab	A	5.61	±	0.02	abc	C
30	5.98	±	0.09	a	AB	5.63	±	0.31	ab	B	6.60	±	0.08	a	A	6.07	±	0.09	a	AB
