# Supplementary material for: Evaluation of Catfish Skin Gelatin-Based Edible Antimicrobial Coating with Lactic Acid and Potassium Sorbate on the Shelf Life and Quality of Fresh Catfish Fillets
Source: Gels. 2026 Jul 2;12(7):584. doi: 10.3390/gels12070584 (PMC13409432; doi:10.3390/gels12070584)
Supplement: Supplementary file 1 [file gels-12-00584-s001.zip › Table S9 and S10 Moisture percentage.pdf]

Table S9. Moisture percentage during 18-day shelf life study of catfish fillets comparing antimicrobial coatings: untreated (C), Potassium sorbate (PS), and Lactic acid (LA). Mean ± Standard Deviation values within each row with different capital letters indicate treatments are significantly different within each day of storage ($p < 0.05$), while different lowercase letters within each column indicate days of storage are significantly different within each individual treatment ($p < 0.05$).

Day	C					LA					PS				
0	81.39%	±	0.68%	a	A	63.58%	±	2.69%	b	B	79.62%	±	1.97%	ab	A
3	76.83%	±	5.29%	a	A	76.79%	±	4.67%	a	A	72.25%	±	7.60%	b	A
6	80.63%	±	2.51%	a	A	75.58%	±	1.49%	a	B	79.03%	±	1.79%	ab	AB
9	79.04%	±	2.53%	a	A	76.35%	±	5.54%	a	A	76.27%	±	1.65%	ab	A
12	80.46%	±	0.70%	a	A	78.87%	±	1.89%	a	A	79.87%	±	0.91%	a	A
15	79.60%	±	1.77%	a	A	79.58%	±	2.89%	a	A	80.39%	±	2.14%	a	A
18	81.25%	±	1.90%	a	A	79.91%	±	2.01%	a	A	79.45%	±	1.35%	ab	A

Table S10. Moisture percentage during 30-day shelf-life study of catfish fillets comparing antimicrobial coatings: untreated (C), Gelatin (G), Gelatin + Lactic acid (G+LA), and Gelatin + Potassium sorbate (G+PS). Mean ± Standard Deviation values within each row with different capital letters indicate treatments are significantly different within each day of storage ($p < 0.05$), while different lowercase letters within each column indicate days of storage are significantly different within each individual treatment ($p < 0.05$).

Day	C					G					G+LA					G+PS				
0	80.9%	±	2.6%	ab	A	79.0%	±	2.8%	a	A	79.0%	±	1.5%	a	A	76.6%	±	4.6%	a	A
3	83.0%	±	0.6%	a	A	80.7%	±	0.9%	a	AB	79.7%	±	0.9%	a	B	79.6%	±	1.9%	a	B
6	80.8%	±	2.1%	ab	AB	81.9%	±	1.1%	a	A	82.0%	±	2.0%	a	A	75.5%	±	5.0%	a	B
9	79.0%	±	1.9%	abc	A	78.9%	±	2.9%	a	A	78.6%	±	3.5%	a	A	77.7%	±	4.2%	a	A
12	81.1%	±	0.3%	ab	A	79.6%	±	0.9%	a	A	79.2%	±	1.2%	a	A	80.3%	±	2.6%	a	A
15	80.6%	±	1.8%	abc	A	81.1%	±	1.3%	a	A	79.5%	±	1.5%	a	A	78.5%	±	2.0%	a	A
18	80.9%	±	2.1%	ab	A	78.6%	±	2.4%	a	A	79.9%	±	1.7%	a	A	76.9%	±	5.1%	a	A
21	75.7%	±	3.1%	c	B	81.8%	±	1.0%	a	A	80.0%	±	3.5%	a	AB	78.7%	±	2.5%	a	AB
24	77.0%	±	2.8%	bc	A	78.8%	±	0.5%	a	A	79.0%	±	2.5%	a	A	79.5%	±	2.9%	a	A
27	78.7%	±	1.0%	abc	A	80.2%	±	0.8%	a	A	79.1%	±	1.9%	a	A	76.5%	±	3.0%	a	A
30	79.3%	±	2.4%	abc	A	77.5%	±	3.7%	a	A	80.2%	±	1.6%	a	A	75.9%	±	3.0%	a	A
